# Supplementary material for: Crossmodal interference on counting performance: Evidence for shared attentional resources
Source: PLoS One. 2023 Nov 10;18(11):e0294057. doi: 10.1371/journal.pone.0294057 (PMC10637692; doi:10.1371/journal.pone.0294057)
Supplement: Attachment — Submitted filename: Response to Reviewers_August_23.docx [file pone.0294057.s004.docx]

Reviewer #1: I would like to thank the authors for their edits. All comments raised in the previous round of review have been addressed. The paper is now ready to be accepted.

We thank you very much for your review work which has allowed us to greatly improve our study.

Reviewer #2: Congratulations again to the authors. This corrected version of the manuscript is worthy of publication. I kindly ask you to adjust just two more minor details. In the METHOD section, in lines 310 and 491, start the sentences with the numerals in full. "Seventy-nine" participants... and "Twenty-six" participants...
All other requests were granted.

We thank you very much for your review work which has allowed us to greatly improve our study. We changed the sentences in lines 310 and 491.
